# The Role of Ranged Horses in Eco-Epidemiology of *Rickettsia raoultii* Infection in China

**DOI:** 10.3389/fmicb.2021.795500

**Published:** 2022-01-17

**Authors:** Qiao-Cheng Chang, Yang Hu, Ting-Ting Wu, Xiao-Xiao Ma, Bao-Gui Jiang, Na Jia, An-Qi Wang, Jia-Fu Jiang

**Affiliations:** ^1^School of Public Health, Shantou University, Shantou, China; ^2^College of Animal Science and Veterinary Medicine, Heilongjiang Bayi Agricultural University, Daqing, China; ^3^Key Laboratory of Zoonosis Research, Ministry of Education, Institute of Zoonosis, College of Veterinary Medicine, Jilin University, Changchun, China; ^4^State Key Laboratory of Pathogen and Biosecurity, Beijing Institute of Microbiology and Epidemiology, Beijing, China; ^5^Animal Health Laboratory, JRU BIPAR ANSES ENVA UPEC, Maisons-Alfort, France

**Keywords:** horse, infection, *Rickettsia raoultii*, tick-borne pathogens, China

## Abstract

*Rickettsia raoultii* is a tick-borne pathogen that infects humans; however, the vertebrate hosts of this pathogen have not been clearly defined. Our molecular examination of *Rickettsia* spp. infecting mammals and ticks in China, identified the *glt*A, *omp*A, and 17KD gene sequences of *R. raoultii* in horses and their ticks. This indicates a role of horses in *R. raoultii* epidemiology.

## Introduction

Tick-borne rickettsioses are recognized as emerging vector-borne infections, infecting both human and animal hosts worldwide. *Rickettsia raoultii* was initially implicated as the causative agent of human infection in 2006 through the detection of DNA in the blood of a Spanish patient (Ibarra et al., 2006), and has since been reported in human infections in many countries of the world; in particular, China (Jia et al., 2014; Li et al., 2018; Dong et al., 2019). *R. raoultii* was first detected in *Dermacentor nuttalli* and *Rhipicephalus pumilio* ticks in 1999 (Rydkina et al., 1999). Although *R. raoultii* has been detected in bloodsucking insects and other tick species, the dominant vectors are generally considered to be *Dermacentor* spp. (Silaghi et al., 2011; Liu et al., 2016).

*Rickettsia raoultii* has been detected in a variety of ticks collected from dogs, cattle, and wildlife (Klitgaard et al., 2017; Chisu et al., 2018; Seo et al., 2020). All of these animals may serve as reservoir hosts for *R. raoultii*; however, experimental evidence is still lacking. Additionally, *R. raoultii* has been detected in squirrels, marbled polecats, red foxes, hedgehogs, yaks, camels and other wild mammals in China (Liu et al., 2018, 2021; Zhao et al., 2019; Li et al., 2020; Fang et al., 2021; Shao et al., 2021). Furthermore, there is a highly significant association of horse contact with tick-borne lymphadenopathy, the pathogens of which are *R. slovaca* and *R. raoultii* (Lakos et al., 2012). Horses also play a role in the epidemiology of Brazilian spotted fever, which is caused by *R. rickettsii*. Horses are considered the most suitable domestic agent for Brazilian spotted fever in some areas (Sangioni et al., 2005), which raises the question of whether horses may also play a role in the disease epidemiology of *R. raoultii*. The findings from this study provide evidence that *Rickettsia* species are in horses and sympatric ticks and that these hosts play a role in the circulation of the spotted fever group of rickettsiae.

## The Study

To identify putative vertebrate hosts of *R. raoultii*, we collected blood samples from horses (*n* = 12), cattle (*n* = 16), sheep (*n* = 10), and brown rats (*Rattus norvegicus*, *n* = 9). All the animals infested by ticks, and all were from the same rangeland in Daqing, northeastern China (46°58′N, 125°03′E), where spotted fever group rickettsiosis was previously reported (Jia et al., 2014). Free-living ticks (*Dermacentor silvarum*, *n* = 96) were collected by the flagging method and engorged adult ticks (*D. silvarum*, *n* = 6) were collected from horses in the same rangeland. Tick eggs (100 eggs, 5 pools) were laid in our laboratory by engorged wild-caught adult female *D. silvarum* from a PCR-positive horse. Larval and nymphal ticks of the same cohort that fed on mice were collected and pooled (35 larvae/10 nymphs; 12 pools in total). All the samples were tested for *Rickettsia* spp. This study was approved by the Research Ethics Committee of Heilongjiang Bayi Agricultural University, China.

Following the manufacturer’s instructions, DNA was extracted from the blood samples and ticks using a Tissue/Blood DNA Extraction Kit (Tiangen Biotech Inc., Beijing, China). PCR was performed to amplify the rickettsial citrate synthase gene (*glt*A), outer membrane protein A-encoding gene (*omp*A), and the 17-kDa antigen-encoding gene fragments; then, sequencing was performed (Li et al., 2018). The sequences obtained in this study were analyzed by an NCBI BLAST search. *Rickettsia* spp. were not detected in any of the samples from cattle, sheep, or brown rats, but sequences most closely related to *R. raoultii* were detected in 6/12 (50%) horses (Table 1). Additionally, *R. raoultii* was detected in 6/6 (100%) engorged ticks, 39/96 (41%) questing, 5/5 (100%) eggs, 5/7 (71%) larvae, and 5/5 (100%) nymphs (Table 1).

**TABLE 1:** The status of *Rickettsia raoultii* infection in different samples.

	Sample	No. tested	No. positive	Prevalence (%)
Vertebrate	Horse	12	6	50
	Cattle	16	0	0
	Sheep	10	0	0
	Wild mice	9	0	0
Tick (*D. Silvarum*)	Adult (engorged)	6	6	100
	Adult (free)	96	39	40.63
	Egg	5	5	100
	Larval	7	5	71.43
	Nymph	5	5	100

**FIGURE 1:**
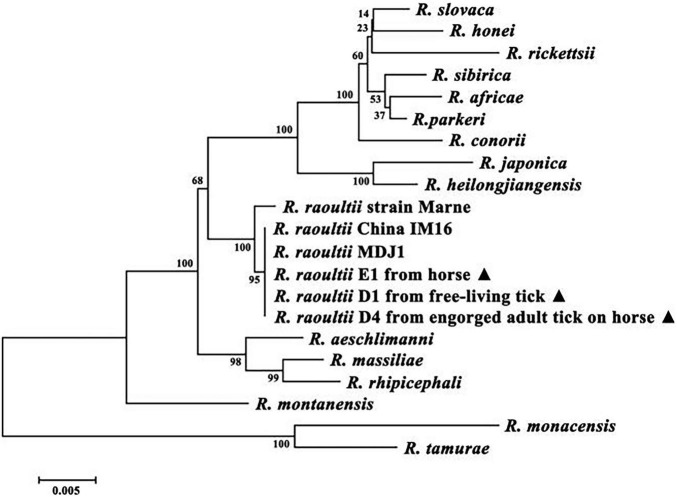
Molecular phylogenetic analysis of *Rickettsia raoultii* isolates from horse. Two genes were concatenated (*glt*A + *omp*A), a total of (1,047 + 498) positions were tested. GenBank accession numbers of the Sequences of the Rickettsia species used to make the concatenated analysis were as follows: *R. rickettsii* (U59729 + U43804), *R. slovaca* (U59725 + U43808), *R. massiliae* (U59719 + U43799), *R. africae* (U59733 + U43790), *R. honei* (U59726 + U43809), *R. conorii* (U59730 + U43806), *R. japonica* (U59724 + U43795), *R. montanensis* (U74756 + U43801), *R. aeschlimanni* (U59722 + U43800), *R. heilongjiangensis* (AF172943 + AF179364), *R. parkeri* (KY124257 + KY271186), *R. rhipicephali* (DQ865206 + DQ865208), *R. monacensis* (LN794217), *R. sibirica* (DQ097081 + DQ097082), *R. tamurae* (AF394896 + DQ103259), *R. raoultii* China IM16 (KY474576 + KY474577), *R. raoultii* strain Khabarovsk (DQ365804 + AH015610), *R. raoultii* strain Marne (DQ365803 + AH015609), *R. raoultii* D1 from free-living tick (MH212179 + MH212186), *R. raoultii* E1 from horse (MH212183 + MH212190) and *R. raoultii* D4 from engorged adult tick on horse (MH212181 + MH212188).

We deposited the sequences from 25 PCR-positive specimens into the GenBank database (accession nos. MH212168–92). For phylogenetic analysis, we used the MegAlign component to perform multiple sequence alignments with the ClustalX1.83 algorithm. A phylogenetic tree was constructed based on the *glt*A and *omp*A sequences identified in this study and other sequences from the GenBank database using the neighbor-joining method. This revealed that the test samples from a horse, questing tick and an engorged adult tick clustered with other *R. raoultii* strains and were most closely related to *R. raoultii* strain MDJ1, which had been isolated from a patient in Mudanjiang, China (Figure 1).

## Discussion

Among our samples, *R. raoultii* was frequently detected in horses, but not in cattle, sheep, or brown rats, despite sharing pasture with the infected horses. A previous study in Xinjiang, China also reported the detection of *R. raoultii* in horses (Li et al., 2020). In this study, horses and other animals residing in the same environment were tested, but positive samples were only detected in horses. The results indicate that horses may be a vertebrate host of *R. raoultii* in the Daqing area. Horses are susceptible to a variety of rickettsial pathogens (Sangioni et al., 2005), and the prevalence rate is higher in some areas (Souza et al., 2016), indicating that horses may well be considered hosts. Therefore, horses may serve as a source of *R. raoultii* infection in ticks. Coincidentally, in a previous study, there were two human cases of *R. raoultii* infection in the same region (Jia et al., 2014).

In this study, the infection rate of *R. raoultii* in *D. silvarum* was 40.63%, which was higher than that (32.25%) in other studies (Wen et al., 2014). Furthermore, all engorged adult ticks and almost all egg/larval/nymphal tick pools were positive for *R. raoultii*. Our results support the idea that *D. silvarum* acts as a vector and a reservoir for *R. raoultii.* Schmuck et al. (2020) confirmed that transovarial transmission might be an efficient way of maintaining the infection cycle of *R. raoultii*, and both transstadial and transovarial transmission of *R. raoultii* have been demonstrated in *D. nuttalli* (Moore et al., 2018).

In conclusion, the main livestock grazing in the area were cattle, horses, and sheep, which were equally likely to be bitten by ticks, but *R. raoultii* was only detected in horses. Horses may therefore serve as a source of *R. raoultii*, contributing to the long-term preservation of *R. raoultii* in this area by promoting the infection of ticks and further increasing the chances of humans becoming infected with *R. raoultii via* tick bites.

## Data Availability Statement

The datasets presented in this study can be found in online repositories. The names of the repository/repositories and accession number(s) can be found below: https://www.ncbi.nlm.nih.gov/genbank/, MH212168-92.

## Author Contributions

J-FJ, A-QW, and Q-CC designed the project and experiments. YH, T-TW, and X-XM conducted the experiments. B-GJ and NJ analyzed the data. YH and Q-CC drafted the manuscript. All authors corrected, edited, and approved the manuscript.

## Conflict of Interest

The authors declare that the research was conducted in the absence of any commercial or financial relationships that could be construed as a potential conflict of interest.

## Publisher’s Note

All claims expressed in this article are solely those of the authors and do not necessarily represent those of their affiliated organizations, or those of the publisher, the editors and the reviewers. Any product that may be evaluated in this article, or claim that may be made by its manufacturer, is not guaranteed or endorsed by the publisher.
